# Influence of LVAD function on mechanical unloading and electromechanical delay: a simulation study

**DOI:** 10.1007/s11517-017-1730-y

**Published:** 2017-11-03

**Authors:** Aulia Khamas Heikhmakhtiar, Ah Jin Ryu, Eun Bo Shim, Kwang-Soup Song, Natalia A. Trayanova, Ki Moo Lim

**Affiliations:** 1 Department of IT Convergence Engineering, Kumoh National Institute of Technology, Yangho-dong, Gumi, Gyeongbuk 730-701 Republic of Korea; 2 Department of Mechanical & Biomedical Engineering, Kangwon National University, Cuncheon, Republic of Korea; 3 Department of Biomedical Engineering, Johns Hopkins University, Baltimore, MD USA

**Keywords:** Ventricular electromechanical model, Heart failure, Calcium transient, Left ventricular assist device

## Abstract

This study hypothesized that a left ventricular assist device (LVAD) shortens the electromechanical delay (EMD) by mechanical unloading. The goal of this study is to examine, by computational modeling, the influence of LVAD on EMD for four heart failure (HF) cases ranging from mild HF to severe HF. We constructed an integrated model of an LVAD-implanted cardiovascular system, then we altered the Ca^2+^ transient magnitude, with scaling factors 1, 0.9, 0.8, and 0.7 representing HF1, HF2, HF3, and HF4, respectively, in order of increasing HF severity. The four HF conditions are classified into two groups. Group one is the four HF conditions without LVAD, and group two is the conditions treated with continuous LVAD pump. The single-cell mechanical responses showed that EMD was prolonged with the higher load. The findings indicated that in group one, the HF-induced Ca2 + transient remodeling prolonged the mechanical activation time (MAT) and decreased the contractile tension, which reduced the left ventricle (LV) pressure, and increased the end-diastolic strain. In group two, LVAD shortened MAT of the ventricles. Furthermore, LVAD reduced the contractile tension, and end-diastolic strain, but increased the aortic pressure. The computational study demonstrated that LVAD shortens EMD by mechanical unloading of the ventricle.

**Electronic supplementary material:**

The online version of this article (10.1007/s11517-017-1730-y) contains supplementary material, which is available to authorized users.

## Introduction

Heart failure (HF) is a chronic and progressive condition, with the heart muscle being unable to pump the appropriate amount of blood to fulfill the needs of the human body [1]. A report from the American Heart Association Statistics Committee and Stroke Statistics Subcommittee concluded that HF is a major cause of morbidity and mortality, and that it contributes significantly to health expenses around the world [16].

A subset of HF includes dyssynchrony between cardiac depolarization and myofiber shortening, which in turn further increases the severity of HF. The time interval between the local myocyte depolarization (electrical activation) and onset of myofiber shortening (mechanical activation) is termed electromechanical delay (EMD) [8]. Normal EMD is typically about 10 ms, and long EMD implies lack of synchrony in cardiac electromechanical activation and a decrease in ventricular pumping efficacy [8].

Constantino et al. [5] identified four major aspects that contribute to prolonged EMD under dyssynchronous HF conditions: remodeled cardiac structure (both heart shape and fiber structure), altered electrical conduction, deranged Ca^2+^ handling, and increased stiffness of the tissue. The timely application of electrical stimulation (termed as cardiac resynchronization therapy (CRT)) can alter the electrical conduction pattern in the ventricles, provide synchrony, and improve the pumping of the heart. The study by Constantino et al. demonstrated that CRT reduced cardiac EMD by reducing the overall electrical activation time [6]. Furthermore, it also found that deranged Ca^2+^ handling resulting in a diminished magnitude of the Ca^2+^ transient, was the primary factor responsible for prolonged EMD. The other three factors had a much smaller contribution to EMD.

An experimental study conducted by Russell et al. in canine and human hearts showed that a mechanical load prolonged the EMD [19]. Although the findings of these studies suggested that EMD decreases if the mechanical load of the ventricles decreases, no research to date has validated this suggestion. A left ventricular assist device (LVAD), used to support cardiac function and improve cardiac output [23], also reduces the mechanical load of the ventricles by enabling an improved pump function. In a previous study of ours, we developed a computational model of the ventricles with LVAD support and showed that the LVAD decreased ventricular after load and improved coronary perfusion [15].

The goal of the present study was to examine, using similar computational modeling, the effect of mechanical load on a single cell and the effect of LVAD on the three-dimensional (3D) distribution of EMD in the four failing heart conditions ranging from mild to severe contractility, and to test the hypothesis that LVAD overall shortens EMD by reducing mechanical afterload. The use of computational modeling overcomes the inability of experimental methodologies to measure and quantify the EMD distribution in the heart.

## Methods

### Model description

**Fig. 1:**
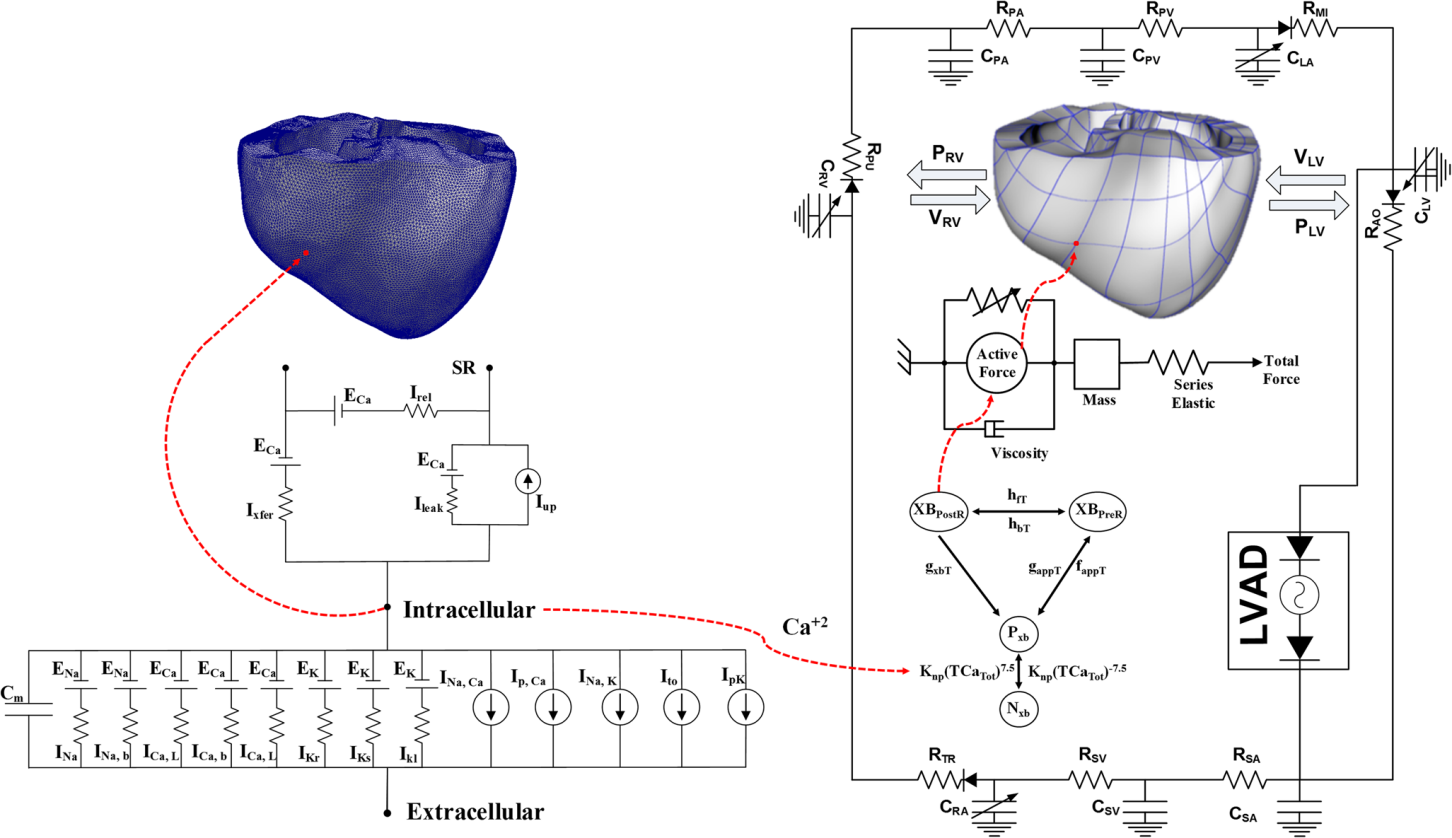
Schematics of the electrical and mechanical elements coupled with calcium transient. *Electrical Element*. It represents the currents, pumps, and exchanger of Ten Tusscher ionic model. Fast inward Na ^+^ current (I _*Na*_), background Na ^+^ current (I _*Na*,*b*_), L-type inward Ca^2+^ current (I _*Ca*,*L*_), background Ca^2+^ current (I _*Ca*,*b*_), rapid delayed rectifier K ^+^ current (I _*Kr*_), slow delayed rectifier K ^+^ current (I _*Ks*_), inward rectifier K _1_ current (I _*K*1_), Na ^+^-Ca^2+^ exchange current (I _*Na*,*Ca*_), sarcoplasmic Ca^2+^ pump current (I _*p*,*Ca*_), Na ^+^-K ^+^ exchange current (I _*Na*,*K*_), transient outward K ^+^ current (I _*to*_), K ^+^ pump current (I _*p*,*K*_), Ca^2+^ release current from the JSR (I _*rel*_), Ca^2+^ leak current from the JSR (I _*rel*_), and Ca^2+^ uptake current into the NSR (I _*up*_). *Mechanical Element*. A schematic diagram of the finite-element ventricular mechanical model coupled with the circulatory and LVAD models. PRV RV pressure, VRV RV volume, PLV LV pressure, VLV LV volume, RPA pulmonary artery resistance, CPA pulmonary artery compliance, RPV pulmonary vein resistance, CPV pulmonary vein compliance, RMI mitral valve resistance, CLA left atrium compliance, RAO aortic valve resistance, RSA systemic artery resistance, CSA systemic artery compliance, RSV systemic vein resistance, CSV systemic vein compliance, RTR tricuspid valve resistance, CRA right atrium compliance, and RPU pulmonary valve resistance

In this study, the 3D image-based electromechanical model of failing ventricles [9, 22] was combined with a lumped model of the circulatory system and LVAD function [15] to construct an integrated model of an LVAD-implanted cardiovascular system. A schematic diagram of the integrated model is shown in Fig. 1. The various components of the combined model are described below. The mathematical equations for the electromechanical model as well as the circulatory system can be found in Suplementary Material.

#### **Electrical model**

The electromechanical model of the failing ventricles used in this study had two dynamic components, namely electrical components and mechanical components, as described in a previous study [9]. The electrical component of the model simulated the propagation of a transmembrane potential wave by solving the monodomain equations on a finite-element tetrahedral mesh comprising 241,725 nodes and 1,298,751 elements. The monodomain partial differential equation (PDE) describes the current flow through ventricles composed of cells connected via conductive gap junctions. The 2D Purkinje network model proposed by Berenfeld and Jalife1 was then mapped onto a 3D endocardial surface of the ventricles [4]. Electrical wave propagation through the Purkinje fiber was implemented by solving the one-dimensional wave propagation equation that triggered the ventricular activation. The current flow in the ventricular tissue was driven by ion exchange across the cellular membranes. These processes were represented using a human ionic model by Ten Tusscher et al. [21], which described the current flow through ion channels, pumps, and exchangers in the myocyte membrane as well as the subcellular Ca cycling as a set of ordinary differential equations (ODEs). Electrical wave propagation in the heart was determined by simultaneously solving the PDE and set of ODEs in the electrical ventricular mesh. The local Ca^2+^ transient at each location in the ventricles served as local trigger for the cardiac mechanics model.

#### **Mechanical model**

The mechanical component of the model simulated ventricular contraction. Ventricular contraction is a result of an active tension generated by the myofilaments of ventricular cells. Ventricular deformation is represented by equations of passive cardiac mechanics, given that the myocardium is an orthotropic (due to the fiber and laminar sheet organization), hyperelastic, and nearly incompressible material with passive mechanical properties defined by an exponential strain-energy function [10]. The model comprised 356 nodes and 172 Hermite elements. The simulation of ventricular contraction was performed by simultaneously solving the active myofilament model equations along with the equations representing passive cardiac mechanics on the finite-element mechanical mesh. The electromechanical model incorporated the biophysical representation of cardiac myofilament dynamics by Rice et al. [18], which represents the excitation-contraction coupling mechanisms (cross-bridge cycling induced by Ca^2+^ release). A set of ODEs and algebraic equations described the Ca^2+^ binding to troponin C, cooperation between regulatory proteins, and cross-bridge cycling. In order to take the HF properties into account, we used a dilated failing ventricle based on a magnetic resonance (MR) image [9, 22] with the size of a kid’s heart, and we scaled the passive scaling constant of the strain-energy function by fivefold to increase the stiffness of the failing myocardium [24]. A lumped-parameter model of the systemic and pulmonic circulatory systems imposed conditions on ventricular volumes and pressure; it was based on a model by Kerckhoffs et al. [13]. We estimated the lumped model with high resistance and with 10% decrease in compliance to support the HF environment.

#### **LVAD function**

This electromechanical model was combined with a lumped model of the circulatory system and an LVAD function based on the study Lim et al. [15] to construct an integrated model of the LVAD-implanted cardiovascular system. The LVAD component was connected to the electromechanical and circulatory models through an inlet in LV, and the outlet was attached to the aorta in the circulatory model. Briefly, the LVAD component was modeled as a flow generator. Constant-flow conditions were used to simulate a continuous LVAD. In this study, the failing ventricle geometry from the MRI has the size of a kid’s heart. Therefore, we adjusted the LVAD flow rate as 50 mL/s as LVAD fully assisted the HF3 and HF4 conditions with this flow rate.

### Simulation procedure

#### **Single-cell simulation**

First, we created four different single-cell myofilament models with different Ca^2+^ transient magnitudes to explore the effect of the altered Ca^2+^ transient, found previously to be the main reason for prolonged EMD under HF conditions (see Section 1), on myocardial force generation, shortening, and ATP consumption. The different Ca^2+^ transient magnitudes represented different levels of severity of HF conditions. The Ca^2+^ transient magnitude was controlled by a scaling factor, with scaling factors 1, 0.9, 0.8, and 0.7 representing HF1, HF2, HF3, and HF4, respectively, in order of increasing HF severity. For each case, the isometric and isotonic contractions of a single-myocardial cell were simulated. To simulate the isometric condition, we set an infinite amount of load to the myofilament model so that the myofilament could not contract. To simulate the isotonic contraction, we set two different load conditions, namely, 30 and 50 mN/mm. The computation was performed for 20 cycles for both isometric and isotonic conditions with a basic cycle length of 600 ms. Comparisons between the isometric and isotonic of 30 mN/mm, and isometric and isotonic of 50 mN/mm were performed at the 20th cycle when the cell had reached a steady-state condition.

#### **3D electromechanics simulation**

First, we simulated the electrical component by inducing an electrical signal to a ventricular tissue through the Purkinje networks with 600 ms for one cycle until steady state. The distribution of the Purkinje networks to the ventricle is based on the experimental study by Durrer et al. [7]. Here, we altered the electrical conduction by adjusting the conduction velocity in the ventricle tissue as 60 cm/s (normally 70 cm/s [12, 20, 21]). The 3D ventricular modeling involved the generation of two groups of models, namely, a control group, which consists of the four HF conditions with no mechanical support and an LVAD group (the four HF conditions treated with LVAD). In a manner similar to the single-myocardial cell simulations, four different Ca^2+^ transient magnitudes were used to simulate mild to severe HF (HF1 to HF4). Then we took the electrical activation time from each node, and set the Ca^2+^ transient information as input to the mechanical simulation. We simulated the mechanical contraction model for 42 s to obtain a steady-state condition with the same cycle length (600 ms). A continuous LVAD with a pumping flow rate of 3 L/min was assumed. Thus, 3D ventricular simulations were executed for eight different conditions, and model responses including spatial distribution of the myocardial tension and contractile adenosine triphosphate (ATP) consumption, strain distribution, and hemodynamic responses (blood pressure, volume, and flow rate) were compared during the last cycle under steady-state condition. The ATP consumption rates were calculated as the product of the cross-bridge detachment rate and single-overlap fraction of thick filaments from each node during the end of systolic volume (ESV) based on the method by Rice et al. [18].

**Fig. 2:**
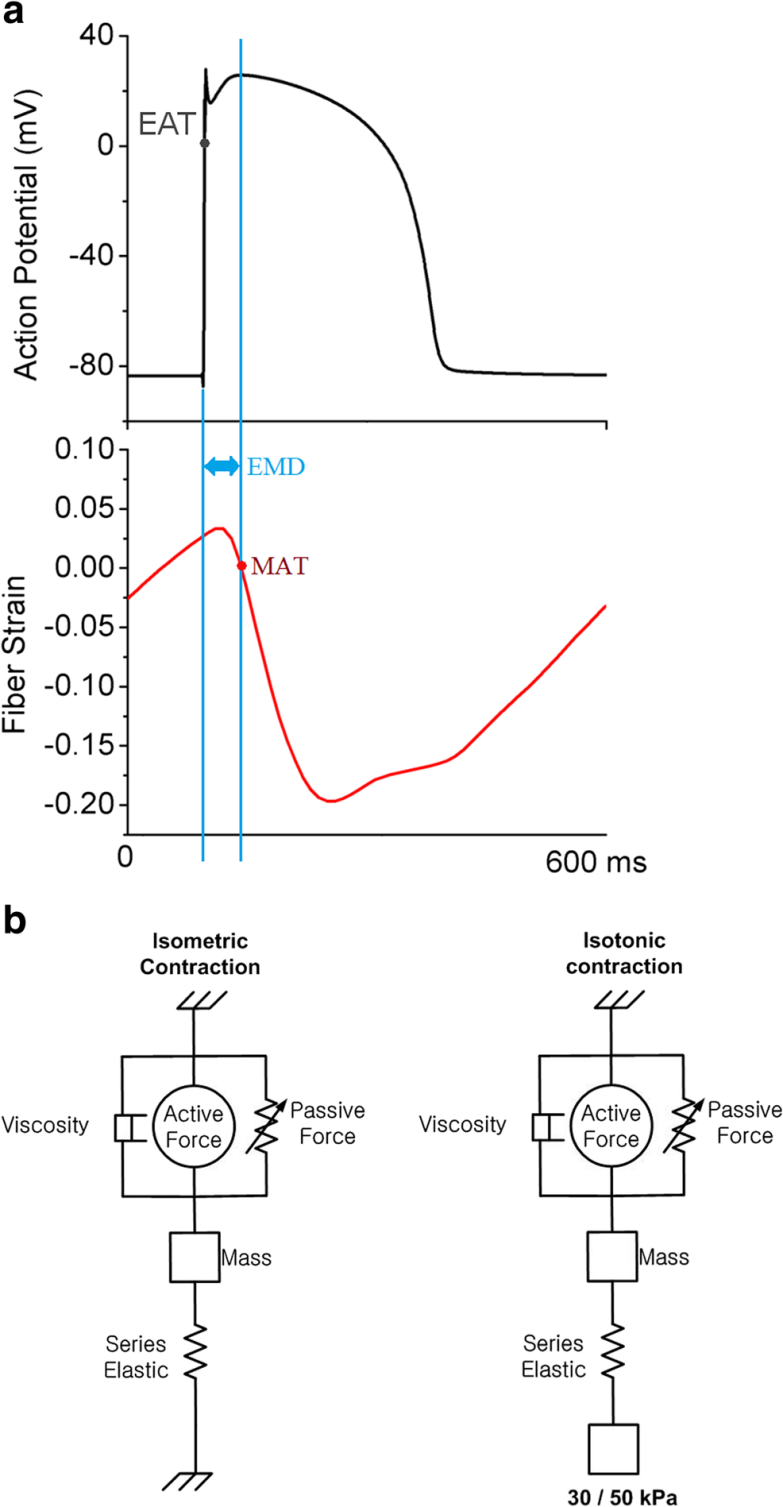
**a** Electrical activation time (EAT) is the time of cardio myocell depolarization (with threshold above 0 mV), mechanical activation time (MAT) is defined as the myofiber shortening at 10%, and electromechanical delay (EMD) is the interval between MAT and EAT. **b** Isometric contraction (left side) is expressed as an active force although the cell does not contract due to the load. Isotonic contraction (right side) is expressed as an active force from the cross bridge, with a specific load (30 or 50 kPa) and forming a contraction

Figure 2a shows the electrical activation time (EAT), mechanical activation time (MAT) and EMD. As in Gurev et al. [8], the as EMD was defined as the time interval between the local myocyte depolarization or EAT and the local myofiber shortening or MAT. The local EAT was defined as the transmembrane voltage exceeding 0 mV, and the onset of myofiber shortening was defined as the time instant at which the myofiber was shortened to 10% of its maximal value. The EMD was obtained by subtracting EAT from MAT at every point in the ventricles. Finally, the 3D distributions of EAT, MAT, and EMD were compared between the control group and the LVAD for the four different levels of HF severity.

## Results

### **Single-cell responses**

**Fig. 3:**
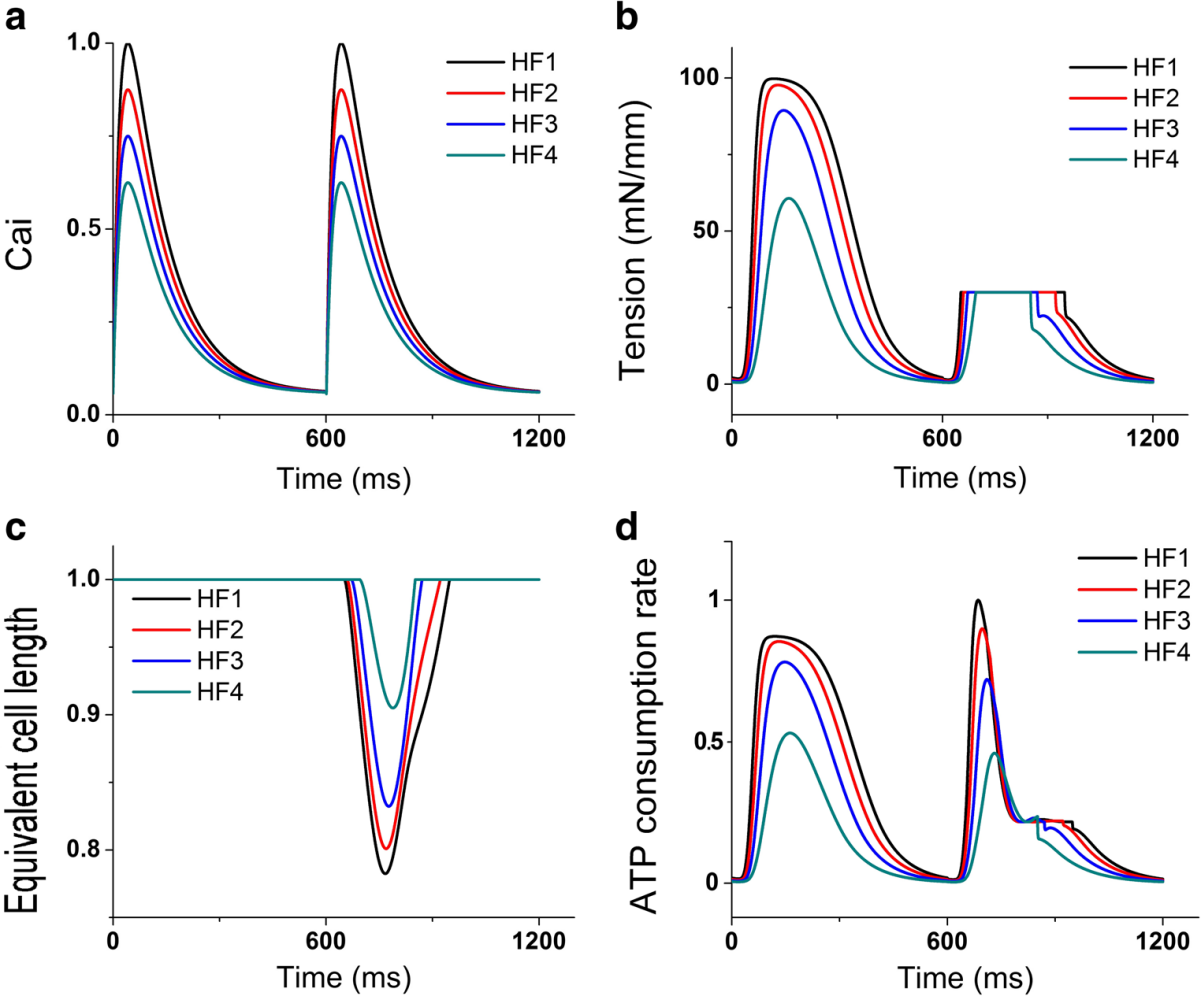
Two cycles of Ca^2+^ transient, myocardial tension (first for isometric contraction and second for isotonic contraction with 30 kPa load), equivalent cell length, and contractile ATP consumption rate for different levels of HF severity (from HF1 to HF4)

**Fig. 4:**
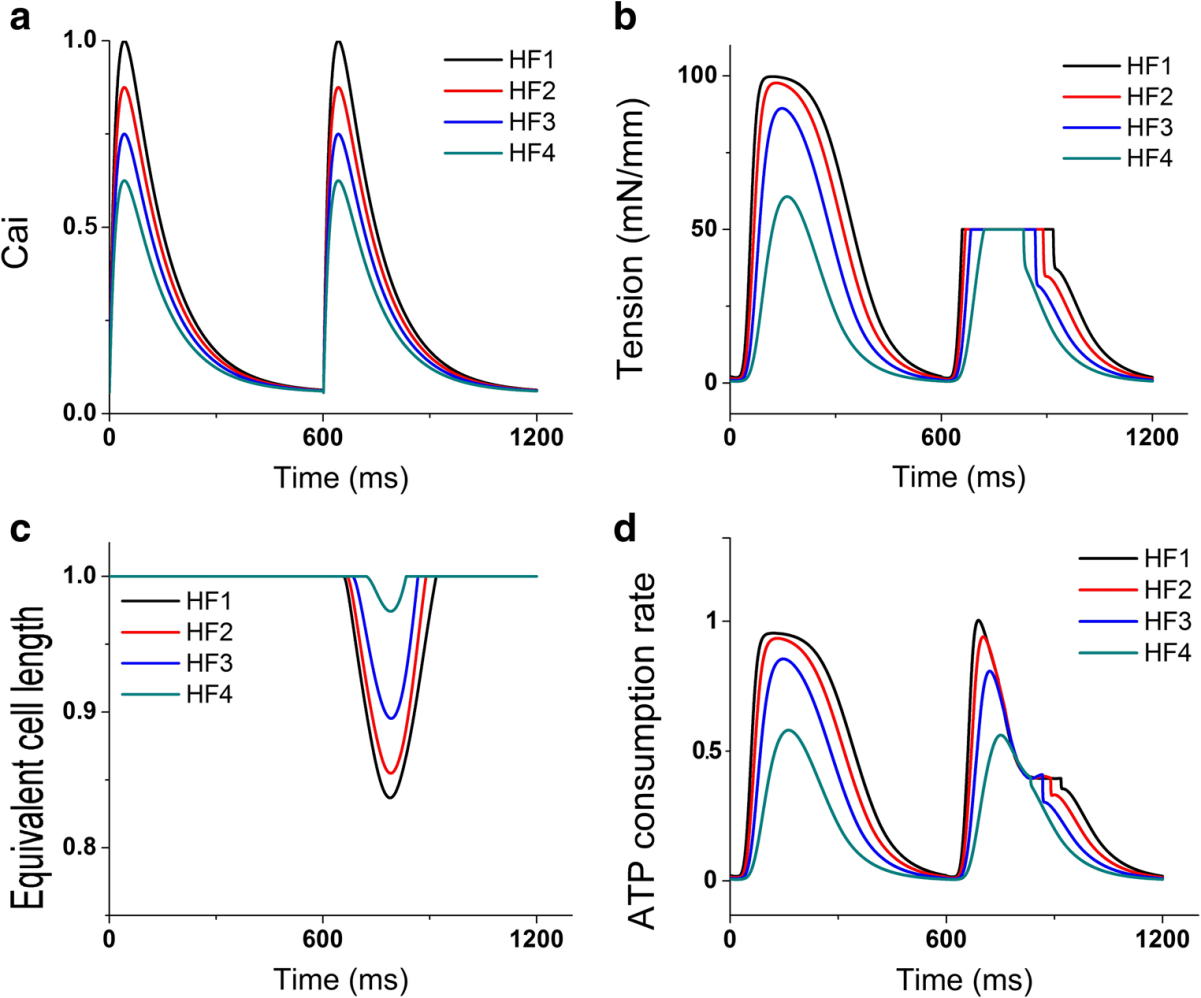
Two cycles of Ca^2+^ transient, myocardial tension (first for isometric contraction and second for isotonic contraction with 50 kPa load), equivalent sarcomere length, and contractile ATP consumption rate for different levels of HF severity (from HF1 to HF4)

Figures 3 and 4 show two cycles of Ca^2+^ transient, myocardial tension, equivalent cell length, and contractile ATP consumption rate for all levels of HF severity (from HF1 to HF4) under 30 and 50 mN/mm load conditions, respectively. The first cycle is a steady-state condition of isometric contraction while the second cycle is the steady state of isotonic contraction. As shown in the figures, HF1 was associated with the highest myocardial tension (Figs. 3b and 4b). It also had the highest ATP consumption rate (Figs. 3d and 4d), and hence, the sarcomere shortened the least amount under the isotonic contraction, compared to that of other HF severity levels (Figs. 3c and 4c). Overall, a greater Ca^2+^ transient magnitude resulted in a higher myocardial tension, shortening amount, and contractile ATP consumption rate. In the isotonic contraction with 30 mN/mm load condition, the shortening values of the sarcomere under the HF2, HF3, and HF4 conditions were 9, 33, and 56% lower compared to that under the HF1 condition, respectively. In the isotonic contraction with the load of 50 mN/mm, the shortening values of the sarcomere under the HF2, HF3, and HF4 conditions were 11, 36, and 84% lower compared to that under the HF1 condition, respectively. Furthermore, the shortening values of the sarcomere with 50 mN/mm load condition were 75, 73, 62, and 27% lower compared to the isotonic contraction with 30 mN/mm load condition for the HF1, HF2, HF3, and HF4 conditions, respectively.

The Ca^2+^ transient magnitude affected not only the tension amplitude, the length shortening, and the ATP consumption rate, but also the time to reach peak values. Tables 1 and 2 show times to reach the peak value of Ca^2+^ transient, myocardial tension, equivalent sarcomere length, and contractile ATP consumption rate for the loads of 30 and 50 mN/mm, respectively. Larger Ca^2+^ transient resulted in faster times-to-peak of myocardial tension, length shortening, and ATP consumption rate. The results were consistent with those obtained by a previous study by Russel et al. [19], which showed that mechanical load contributed to prolonged EMD. We also compared the sarcomere shortening time-to-peak during isotonic contraction with loads of 30 and 50 mN/mm. In HF1 and HF2, the cell with a load of 30 mN/mm shortened 11.7 and 11.6% faster, respectively, than a cell with load 50 mN/mm. In HF3, the cell with a load of 30 mN/mm shortened 7.4% faster when compared with the cell with a load of 50 mN/mm. However, in HF4, the sarcomeres of both cells shortened the same amount.

**Table 1:** Single-cell peak time value of Ca, tension, equivalent cell length, and ATP with 30 kPa load (ms)

HF level	Ca^2+^		Tension		Equivalent cell length		ATP	
	Isometric	Isotonic	Isometric	Isotonic	Isometric	Isotonic	Isometric	Isotonic
HF1	41	41	115	52	0	166	114	85
HF2	41	41	130	60	0	167	127	96
HF3	41	41	146	72	0	175	147	110
HF4	41	41	161	95	0	188	161	129

**Table 2:** Single-cell peak time value of Ca, tension, equivalent cell length, and ATP with 50 kPa load (ms)

HF level	Ca^2+^		Tension		Equivalent cell length		ATP	
	Isometric	Isotonic	Isometric	Isotonic	Isometric	Isotonic	Isometric	Isotonic
HF1	41	41	115	59	0	188	114	88
HF2	41	41	130	68	0	189	127	101
HF3	41	41	146	84	0	189	147	119
HF4	41	41	161	121	0	188	161	149

### **3D ventricular responses**

**Fig. 5:**
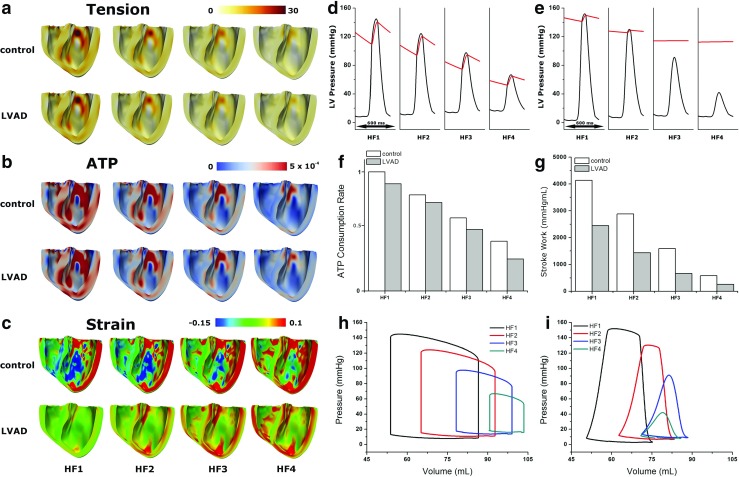
**a** Tension at end of systole, **b** ATP at end of systole, **c** strain at the end of diastole, **d** LV pressure (black lines) and aortic pressure (red lines) of the control group, **e** LV pressure (black lines) and aortic pressure (red lines) in the LVAD group, **f** energy consumption in form of the ATP taken from one cycle, **g** stroke work, **h** pressure-volume loop of control group, and **i** pressure-volume loop in the LVAD group

Figure 5 shows the calculated transmural distribution of myocardial tension (Fig. 5a), contractile ATP consumption rate (Fig. 5b), and strain (Fig. 5c), as well as the pressure waveforms of LV and aorta as a function of time (Fig. 5d is for the control group and Fig. 5e for the LVAD therapy group), and the overall ATP consumption rate of all nodes in one cycle (Fig. 5f), LV stroke work (Fig. 5g), and LV pressure-volume loop (Fig. 5h refers to the control group and Fig. 5i to the LVAD therapy group) for all HF severities. The myocardial tension and contractile ATP consumption rate distribution were obtained at end-systole, while the strain distribution was obtained at end-diastole.

The results indicated that tension and ATP consumption rate were decreased in the LVAD and control groups following the decrease in Ca^2+^ transient magnitude. For the same HF severity condition, LVAD decreased tension and ATP consumption (Fig. 5a and b). However, strain increased when Ca^2+^ transient was decreased. The LVAD treatment reduced ventricular strain for severe HF (Fig. 5c).

Figure 5d, e shows the steady-state responses of the LV pressure (black lines) and aortic pressure (red lines) in the control and LVAD groups, respectively. As illustrated in Fig. 5d, under the HF1 condition, the LV peak pressure was 145 mmHg, and the aortic pressure was between 109 and 140 mmHg. Under the HF2 condition, these numbers were 124 mmHg, and between 94 and 120 mmHg, respectively. In the HF3 condition, the LV peak pressure was 98 mmHg, and the aortic pressure ranged between 74 and 95 mmHg. The HF4 condition resulted in the lowest LV and aortic peak pressures, with the LV peak pressure reaching 67 mmHg, and the aortic pressure being between 65 and 52 mmHg. Additionally, during the diastolic phase, the LV pressure progressively increased from HF1 to HF4.

As Fig. 5e indicates, LVAD fully assisted the LV in pumping blood in the HF3 and HF4 conditions, but for the HF1 and HF2 conditions, it provided only partial assistance. In the HF1 and HF2 conditions, the LV peak pressures were 152 and 130 mmHg, respectively. The aortic pressures ranged between 141 and 152 mmHg for the HF1 condition, and between 126 and 129 mmHg for the HF2 condition. In the HF3 and HF4 conditions, the LV peak pressures were 91 and 42 mmHg, respectively. However, the aortic pressures remain the same for both the HF3 and HF4 conditions at 113 mmHg.

The reason why the LVAD did not fully assist in the distribution of blood under conditions HF1 and HF2 was because the LV pressures in these cases exceeded the aortic pressures during the systolic phase. Under HF3 and HF4 conditions, the blood volume from the LV was fully unloaded by the LVAD and transported directly toward the aorta by passing it from the aortic valve. The aortic pressure under HF4 was the same as under HF3 (113 mmHg). The LVAD function not only reduced the LV pressure by mechanical unloading, but also maintained sufficient aortic pressure to support the peripheral and coronary arteries.

Figure 5f shows the overall ATP consumption rate during a single heart beat in both LVAD and control groups under HF1, HF2, HF3, and HF4 conditions. Generally, a more severely failing ventricle consumed less ATP for myocardial shortening. Furthermore, the LVAD group consumed lower ATP when compared to the control group (Fig. 5f). Specifically, LVAD therapy reduced contractile ATP consumption by 10% in HF1, 8% in HF2, 17% in HF3, and 35% in HF4. These results demonstrated that LVAD reduced the LV mechanical load and contractile energy consumption, especially under severe HF.

Figure 5g presents LV stroke works for both control and LVAD groups across all HF levels. In a manner similar to the data for the overall ATP consumption rate, the more severely failing ventricle performed less stroke work to pump blood. Additionally, the LVAD group performed less stroke work as compared to the control group (Fig. 5g). This was because LVAD assisted the LV to pump blood into the aorta. The LVAD therapy reduced LV stroke work by 60% in HF1, 50% in HF2, 42% in HF3, and 77% in HF4.

Figure 5h and i present LV pressure-volume loops in the control and LVAD groups for all the HF severities. The pressure-volume loops were shifted to the right as HF severity increased, and shifted back to the left following LVAD therapy. The pressure decreased as Ca^2+^ transient magnitude diminished which makes less contractility. Tables 3 and 4 show end of diastolic volume (EDV), ESV, stroke volume (SV), and ejection fraction (EF) as obtained from the pressure-volume loops for all HF severities for control and LVAD groups, respectively.

**Table 3:** EDV, ESV, SV, and EF of control groups (in mL except for EF)

Control	EDV	ESV	SV	EF
HF1	87	54	33	40%
HF2	93	65	28	30%
HF3	100	78	22	22%
HF4	104	91	13	13%

**Table 4:** EDV, ESV, SV, and EF of LVAD groups (in mL except for EF)

LVAD	EDV	ESV	SV	EF
HF1	75	51	24	32%
HF2	83	63	20	24%
HF3	88	71	0	Fully assisted
HF4	86	71	0	Fully assisted

**Fig. 6:**
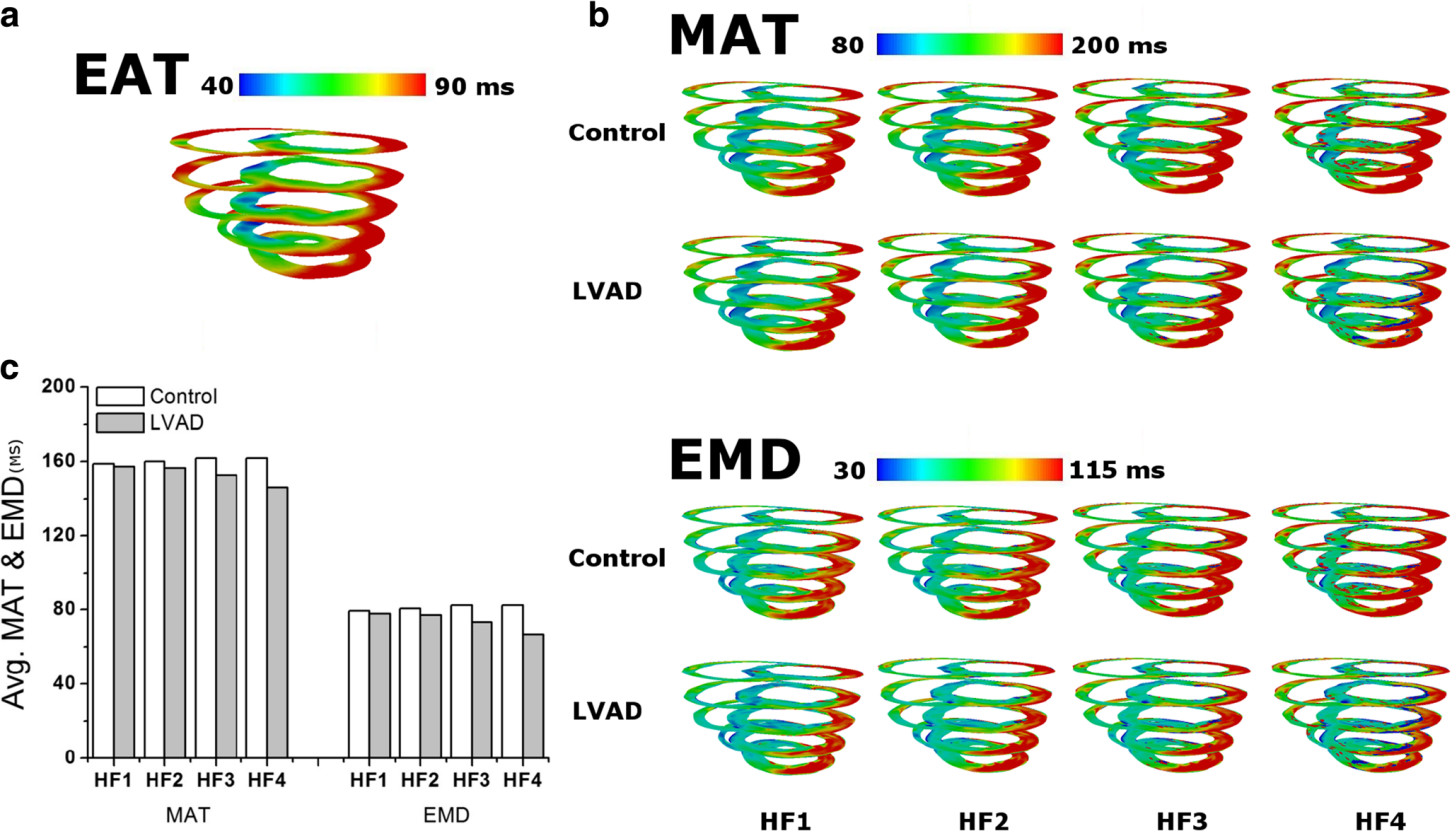
**a** 3D distribution of electrical activation time taken when the membrane potential of each node is above 0; **b** mechanical activation time based on 10% strain shortening and electromechanical delay, which is the interval time of EAT and MAT; (**c**) and average time of MAT and EMD in HF1, HF2, HF3, and HF4

Figure 6 shows the transmural distribution of electrical activation time (Fig. 6a), and MAT and EMD (Fig. 6b). It also shows the average time of MAT and EMD throughout the entire ventricles for different HF severity in LVAD and control groups (Fig. 6c). Overall, MATs were prolonged for increasing severity of HF while all the EATs were constant. Therefore, more severe HF resulted in longer EMDs (Fig. 6b). The spatial average of MATs was 159 ms in HF1, 160 ms in HF2, 162 ms in HF3, and 163 ms in HF4. Therefore, the spatial average of EMDs was 79, 81, 82, and 83 ms, respectively. LVAD therapy reduced MAT at each severity level and thereby also reduced EMD. The LVAD therapy reduced the average MAT by 1% in HF1, 2% in HF2, 3% in HF3, and 6% in HF4. Therefore, average EMDs were reduced by 1, 2, 4, and 18%, respectively. Results indicated that both MAT and EMD were reduced by the mechanical unloading even under mild HF conditions (HF1) in the control group. Thus, LVAD reduced MAT and EMD in all cases.

## Discussion

This study used a sophisticated computational approach (Fig. 1) to predict the effect of Ca^2+^ remodeling and LVAD function on the cardiac unloading and electromechanical delay in a three-dimensional space of an image-based failing ventricle. In order to take the HF properties into account, we reduced the electrical conductivities, increased the myocardial stiffness, and remodeled the calcium transient. In this study, we mainly vary the Ca^2+^ transient by scaling its magnitude from 1 to 0.7 to mimic the varying HF severities characterized by systolic dysfunction. Despite other remodeling in HF, Constantino et al. [5] reported that primary reason for the prolonged EMD was deranged Ca^2+^ handling; hence, we varied only the Ca^2+^ transient magnitude. For each level of HF, two groups were considered: an LVAD treatment group and a control group. We also quantified how the load prolonged the mechanical contraction in four HF severities by a single-cell simulation. Major variables of electromechanical responses were observed including myocardial tension, ATP consumption, strain, LV pressure, aortic pressure, EF, MAT, and EMD. The following were the main findings of this study:

1. Failure in the cardiac myocyte induced by Ca^2+^ transient remodeling prolonged MAT and EMD (Figs. 3, 4, and 6);
2. The mechanical load prolonged MAT and EMD (Figs. 3 and 4);
3. The remodeled Ca^2+^ transient prolonged MAT throughout the ventricles (Fig. 6). It also reduced the contractile tension, which reduced the LV and aortic pressures, ATP consumption rate, and LV stroke work and increased the end-diastolic strain (Fig. 5);
4. LVAD shortened MAT (hence, EMD as well) throughout the ventricles (Fig. 6) and reduced the contractile tension, which reduced the LV pressure but increased the aortic pressure, ATP consumption rate, LV stroke work, and end-diastolic strain (Fig. 5).

Several clinically significant responses such as ATP consumption rate, tension, equivalent cell length, LV and aortic pressures, LV pressure-volume diagram, and LV stroke work were examined. We found that these responses worsened with HF severity. However, as we hypothesized here, improvements in ventricular pumping performance due to LVAD were even higher under more severe HF conditions. The results indicate that even though LVAD improved the heart pumping, the peak of LV pressure in the LVAD group was slightly higher compared to that of the control group in the case of HF1, which represented a mild HF condition.

A computational study by Yu et al. showed that CRT reduced the ATP consumption by up to 20% by pacing in the anterior between the apex and base in left bundle branch block (LBBB) condition [11]. This study showed that LVAD reduced the ATP consumption by up to 35% in severe HF. Therefore, LVAD treatment in combination with CRT can be considered for patients who suffer severe DHF in order to reduce the ventricular energetic load.

EMD as well as MAT increased as the HF severity increased. An increase in the HF severity lowered the Ca^2+^ concentration in the cardiac muscle, resulting in longer MAT and EMD. LVAD reduced MAT and EMD via mechanical unloading in all HF cases (Fig. 6). LVAD also reduced the ventricular pressure, volume, tension, and ATP consumption (Fig. 5). In contrast to the longer EMD under more severe HF conditions, LVAD performed better by providing a larger reduction in MAT and EMD due to the higher reduction in mechanical unloading.

The results indicated that the differences in the cardiac responses between LVAD and control groups were not significant under mild HF conditions. Thus, the heart does not necessarily need LVAD under normal conditions. By contrast, under the most severe HF, the LV and aorta pressures, energy consumption, stroke work, tension, strain activation, MAT, and EMD showed significant improvements under LVAD treatment.

There are some limitations of this study. In our electromechanical model of a failing ventricle, we did not consider the coronary circulation to simplify the computation. We also did not consider the long-term recovery effect of LVAD to the ventricle, whereas previous studies have found that LVAD assists the HF recovery [2, 3, 14, 17]. It may be necessary to consider additional HF properties to quantify the LVAD contribution to EMD under various HF conditions in the future. For instance, a subset of HF in which the heart exhibits dyssynchrony contractions due to the LBBB could be a useful variation. In addition, the integration of autonomic nervous system model for peripheral vascular resistance dynamic would also have a significant impact to examine the correlation between HF and the peripheral vascular system. Nevertheless, the findings of this study present a first step in quantifying the effect of mechanical unloading on cardiac EMD.

## Conclusions

In conclusion, LVAD shortens EMD by mechanical unloading in mild HF, and its performance increases with the severity of the HF. This computational study validated the hypothesis that the LVAD can shorten EMD by mechanical unloading in the ventricle.

## Electronic supplementary material

Below is the link to the electronic supplementary material.

(DOC 19.4 KB)
