# Supplementary material for: Influence of LVAD function on mechanical unloading and electromechanical delay: a simulation study
Source: Med Biol Eng Comput. 2017 Nov 3;56(5):911–21. doi: 10.1007/s11517-017-1730-y (PMC5906510; doi:10.1007/s11517-017-1730-y)
Supplement: Supplementary file 1 — (DOC 19.4 KB) [file 11517_2017_1730_MOESM1_ESM.docx]

**Supplementary Material**

**Mathematical equation for the electrophysiological model**

To simulate the cardio-electrophysiological behavior, we used a mathematical model of the human ventricle based on the study by ten Tusscher et al. [22].

The ionic movement across the cell membrane of the myocardium through the ionic channels can be expressed by the following electrical circuit model:

$\frac{dV}{dt}=\frac{-I_{ion}+I_{stim}}{C_{m}}$ (1)

where *V* (mV) is voltage difference, *t* (ms) is time, *I_ion_* is major ionic currents, *I_stim_* is stimulus current, and *Cm* is capacitance per unit of surface area. Equation (1) represents the electrophysiology of a single cell with stimulus current. *I_ion_* consists of the major ionic currents such as sodium (Na^+^), potassium (K^+^), and calcium (Ca^2+^) as follows:

$I_{ion}=I_{Na}+I_{K1}+I_{to}+I_{Kr}+I_{Ks}+I_{CaL}+I_{NaCa}+I_{NaK}+I_{pCa}+I_{pK}+I_{bCa}+I_{bNa}$ (2)

To simulate the electrophysiological propagation in a three-dimensional ventricular model, the Eq. (1) of the single-cell can be expanded by the following partial differential equation (PDE):

$\frac{dV}{dt}=\frac{-I_{ion}+I_{stim}}{C_{m}}+\frac{1}{\rho_{x}S_{x}C_{m}}\frac{\partial^{2}V}{\partial x^{2}}+\frac{1}{\rho_{y}S_{y}C_{m}}\frac{\partial^{2}V}{\partial y^{2}}+\frac{1}{\rho_{z}S_{z}C_{m}}\frac{\partial^{2}V}{\partial z^{2}}$ (3)

where *ρ* is cellular resistivity and *S* is surface to volume ratio in *x*, *y*, and *z* directions.

The equations of the currents that comprise *I_ion_* are as follows:

Fast sodium current

$I_{Na}=G_{\mathrm{Na}}m^{3}hj(V-E_{\mathrm{Na}})$ (4)

Inward rectifier potassium current

$I_{K1}=G_{K1}\sqrt{\frac{K_{o}}{5.4}}x_{K1\infty}(V-E_{K})$ (5)

Transient outward current

$I_{\mathrm{to}}=G_{\mathrm{to}}rs(V-E_{K})$ (6)

Rapid delay rectifier current

$I_{\mathrm{Kr}}=G_{\mathrm{Kr}}\sqrt{\frac{K_{o}}{5.4}}x_{r1}x_{r2}(V-E_{K})$ (7)

Slow delay rectifier current

$I_{\mathrm{Ks}}=G_{\mathrm{Ks}}x_{s}^{2}(V-E_{\mathrm{Ks}})$ (8)

L-type calcium current

$I_{\mathrm{CaL}}=G_{\mathrm{CaL}}{dff}_{CaL}4\frac{{VF}^{2}}{RT}\frac{\mathrm{Ca}_{i}e^{2V/\mathrm{RT}}-0.341\mathrm{Ca}_{o}}{e^{2V/\mathrm{RT}}-1}$ (9)

Sodium/calcium exchange current

$I_{\mathrm{NaCa}}=K_{\mathrm{NaCa}}\frac{e^{\gamma VF/\mathrm{RT}}\mathrm{Na}_{i}^{3}\mathrm{Ca}_{o}-e^{\left( \gamma-1 \right)VF/\mathrm{RT}}\mathrm{Na}_{o}^{3}\mathrm{Ca}_{i}\alpha}{(K_{\mathrm{mNai}}^{3}+\mathrm{Na}_{o}^{3})(K_{mCa}+\mathrm{Ca}_{o})(1+k_{sat}e^{\left( \gamma-1 \right)VF/\mathrm{RT}})}$ (10)

Pump current of sodium/potassium, calcium, and potassium

$I_{\mathrm{NaK}}=P_{\mathrm{NaK}}\frac{K_{o}\mathrm{Na}_{i}}{(K_{o}+K_{mK})({Na}_{i}+K_{mNa})(1+{0.1245e}^{-0.1VF/\mathrm{RT}}+{0.0353e}^{-VF/\mathrm{RT}})}$ (11)

$I_{\mathrm{pCa}}=G_{\mathrm{pCa}}\frac{\mathrm{Ca}_{i}}{K_{\mathrm{pCa}}+\mathrm{Ca}_{i}}$ (12)

$I_{\mathrm{pK}}=G_{\mathrm{pK}}\frac{V-E_{K}}{1+e^{(25-V)/5.98}}$ (13)

Background currents

$I_{\mathrm{bNa}}=G_{\mathrm{bNa}}(V-E_{\mathrm{Na}})$ (14)

$I_{\mathrm{bCa}}=G_{\mathrm{bCa}}(V-E_{\mathrm{Ca}})$ (15)

Calcium dynamics

$I_{leak}=V_{leak}\left( {Ca}_{sr}-{Ca}_{i} \right)$ (16)

$I_{up}=\frac{V_{maxup}}{1+{K_{up}^{2}}/{{Ca}_{i}^{2}}}$ (17)

$I_{rel}=\left( a_{rel}\frac{{Ca}_{sr}^{2}}{b_{rel}^{2}+{Ca}_{sr}^{2}}+C_{rel} \right)dg$ (18)

${Ca}_{ibufc}=\frac{{Ca}_{i}\times{Buf}_{c}}{{Ca}_{i}+K_{bufc}}$ (19)

$\frac{{dCa}_{itotal}}{dt}=\frac{-I_{Cal}+I_{bCa}+I_{pCa}-{2I}_{NaCa}}{{2V}_{c}F}+I_{leak}-I_{up}+I_{rel}$ (20)

${Ca}_{srbufsr}=\frac{{Ca}_{sr}\times{Buf}_{sr}}{{Ca}_{sr}+K_{bufsr}}$ (21)

$\frac{{dCa}_{stotal}}{dt}=\frac{V_{C}}{V_{sr}}\left( -I_{leak}+I_{up}-I_{rel} \right)$ (22)

The calcium dynamic is used as input to the mechanical compartment. For a better description of the equation, please see the paper by ten Tusscher et al. [22].

**Mathematical equation for the myofilament or mechanical model**

In this study, we used the cardio myofilament model proposed by Rice et al. [20]. Generally, they used ordinary differential equations (ODEs) to simulate the change in sarcomere length (*SL*). For the isometric contraction, *dSL*/*dt* = 0, and *SL* is assumed to remain in the original state, *SL*_0_. For the isotonic contraction, the following equation is used to calculate *SL*:

$\frac{d}{dt}SL=\frac{{Integral}_{Force}+\left( {SL}_{0}-SL \right)\times viscosity}{mass}$ (23)

${Integral}_{Force}=\int_{0}^{t} \left( F_{active}\left( x \right)+F_{passive}\left( x \right)-F_{preload}-F_{afterload}\left( x \right) \right)dt$ (24)

$F_{afterload}\left( x \right)=KSE\times\left( x-{SL}_{0} \right)$ (25)

where KSE is the stiffness in normalized units of F/mm and x is the sarcomere length.

**Mathematical equation for the lumped model**

The lumped model in this study follows the model by Kerckhoffs et al. [15], which is shown on the right side in Fig. 1. The equations of the components are shown as follows:

$-R_{\mathrm{SA}}\dot{Q}_{SA}+\frac{1}{C_{SA}}Q_{SA}=V_{SV}$ (26)

$-R_{\mathrm{SV}}\dot{Q}_{SV}+\frac{1}{C_{SV}}Q_{SV}=V_{RA}$ (27)

$-R_{\mathrm{RA}}\dot{Q}_{RA}+\frac{1}{C_{RA}}Q_{RA}=V_{RV}$ (28)

$-R_{\mathrm{RV}}\dot{Q}_{RV}+\frac{1}{C_{RV}}Q_{RV}=V_{PA}$ (29)

$-R_{\mathrm{PA}}\dot{Q}_{PA}+\frac{1}{C_{PA}}Q_{PA}=V_{PV}$ (30)

$-R_{\mathrm{PV}}\dot{Q}_{PV}+\frac{1}{C_{PV}}Q_{PV}=V_{LA}$ (31)

$-R_{\mathrm{LA}}\dot{Q}_{LA}+\frac{1}{C_{LA}}Q_{LA}=V_{LV}$ (32)

$-R_{\mathrm{LV}}\dot{Q}_{LV}+\frac{1}{C_{LV}}Q_{LV}=V_{SA}$ (33)

where *R* is resistance, *Q* is flux, *V* is volume, *C* is compliacne, *SA* is systemic artery, *SV* is systemic vein, *RA* is right atrium, *RV* is right ventricle, *PA* is pulmonary artery, *PV* is pulmonary vein, *LA* is left atrium, and *LV* is left ventricle. The subscript symbols represent each compartment of the circulatory.
